# A negative feedback loop underlies the Warburg effect

**DOI:** 10.1038/s41540-024-00377-x

**Published:** 2024-05-24

**Authors:** Alok Jaiswal, Raghvendra Singh

**Affiliations:** Department of Chemical Engineering, Indian Institute of Technology Kanpur, Kanpur, 208016 India

**Keywords:** Systems biology, Cancer

## Abstract

Aerobic glycolysis, or the Warburg effect, is used by cancer cells for proliferation while producing lactate. Although lactate production has wide implications for cancer progression, it is not known how this effect increases cell proliferation and relates to oxidative phosphorylation. Here, we elucidate that a negative feedback loop (NFL) is responsible for the Warburg effect. Further, we show that aerobic glycolysis works as an amplifier of oxidative phosphorylation. On the other hand, quiescence is an important property of cancer stem cells. Based on the NFL, we show that both aerobic glycolysis and oxidative phosphorylation, playing a synergistic role, are required to achieve cell quiescence. Further, our results suggest that the cells in their hypoxic niche are highly proliferative yet close to attaining quiescence by increasing their NADH/NAD+ ratio through the severity of hypoxia. The findings of this study can help in a better understanding of the link among metabolism, cell cycle, carcinogenesis, and stemness.

## Introduction

Otto Heinrich Warburg found that, unlike normal cells, cancer cells use incomplete glycolysis and ferment glucose into lactate^1^, even in the presence of oxygen, to proliferate in place of the complete oxidation of glucose using the TCA cycle and oxidative phosphorylation. A definite understanding of the Warburg effect^2^ was not known because complete oxidation appeared to be more efficient than incomplete glycolysis^1^ since the latter involves the wastage of glucose carbon excreted as lactate^3^. Nonetheless, aerobic glycolysis is a general feature of the regulation of cell proliferation both in cancer and normal cells^3^.

In glycolysis, glucose is oxidized to glyceraldehyde 3-phosphate, which is oxidized to 1,3-bisphosphoglycerate by the enzyme glyceraldehyde 3-phosphate dehydrogenase (GAPDH)^3^. Further, in this process, NADH is produced, although NAD+ is required for the GAPDH activity^3^ to carry on the glycolysis. Thus, in the cytoplasm, NAD+ needs to be regenerated from NADH. There are three ways NAD+ can be regenerated from NADH in the cytosol: 1. The malate-aspartate shuttle (MAS) 2. The glycerol 3-phosphate shuttle (G3PS) 3. The reduction of pyruvate to lactate by lactate dehydrogenase (LDH)^3^. MAS and G3PS help translocate the reducing power of glucose to oxidative phosphorylation in the mitochondria^3^. In contrast, the reduction of pyruvate to lactate does not participate in the running of oxidative phosphorylation^3^. Recently, it has been found that the driving force for aerobic glycolysis is the saturation of the NADH shuttles of MAS and G3PS toward oxidative phosphorylation^3^. In contrast, our model and analysis suggest a synergistic relation between aerobic glycolysis and oxidative phosphorylation.

Cancer cells have a high glycolysis rate, which occurs when the NADH/NAD+ ratio is low because a high NADH/NAD+ ratio inhibits GAPDH^4^. Further, NAD+ is produced from NADH by the cytosolic malate dehydrogenase 1 (MDH1)^5^, decreasing NADH/NAD+ ratio in the cytoplasm. Furthermore, human tumors overexpress MDH1, and the MDH1 expression correlates with poor prognosis^5^, suggesting a role of the decreased NADH/NAD+ ratio in tumor progression.

**Fig. 1:**
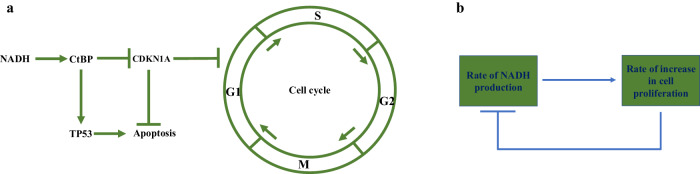
NADH controls cell cycle and cell proliferation. **a** The regulation of cell cycle and apoptosis by NADH through CtBP. **b** The negative feedback loop between the rate of NADH production and the rate of increase in cell proliferation.

BRCA1 transactivates the cyclin-dependent kinase inhibitor CDKN1A^6^. On the other hand, CtBP, through the CtIP-CtBP-BRCA1 repressor complex causes CDKN1A promoter deacetylation, repressing CDKN1A transcription and promoting the G1-S transition of cells^7^. Further, NADH is required for the CtBP dimerization and the formation of the repression complex involving the dimeric CtBP^8^. Thus, NADH promotes the cell cycle progression through the dimeric CtBP by repressing CDKN1A. Besides the cell cycle, a link between aerobic glycolysis, NADH-CtBP, and TP53 has been established^4^. NADH, through the dimeric CtBP, causes TP53 accumulation^4^. Thus, an increase in NADH concentration may cause apoptosis through TP53 accumulation. Similarly, a decrease in CDKN1A, which can be caused by the repression of CDKN1A by NADH-CtBP, may induce apoptosis^9^_._ Thus, NADH causes both cell cycle progression and apoptosis (Fig. 1a). Further, one of the reasons why cancer cells supplement oxidative phosphorylation with aerobic glycolysis is that the cytoplasmic NADH directly controls the cell cycle and apoptosis, the processes that control cell proliferation directly through the repression of CDKN1A and accumulation of TP53 by NADH-CtBP.

However, how glycolysis causes cell proliferation while producing lactate lacked clarity. Our results show that a reduction in NADH/NAD+ ratio, while NADH-CtBP causes the cell cycle progression, increases cell proliferation. This is due to a negative feedback loop (NFL) between the rate of increase in cell proliferation and the rate of increase in cytoplasmic NADH concentration (Fig. 1b), generating an inverse relationship between NADH and cell proliferation (Eq. 17, Methods section). Further, the results show that cell proliferation is higher if either the effectiveness of NADH increase in the cytoplasm through glycolysis is lower and/or pyruvate-to-lactate conversion is higher and/or flux of NADH and NADH-equivalent to mitochondria is higher. Thus, retention of the reducing power of NADH in the cytoplasm is detrimental to cell proliferation, even though NADH is involved in cell cycle progression. Further, we show that oxidative phosphorylation and aerobic glycolysis have a synergistic relation. Furthermore, aerobic glycolysis serves as an amplifier of oxidative phosphorylation in increasing cell proliferation when NADH/NAD+ ratio decreases and in keeping a high concentration of NAD+ in cells. The paper is organized as follows:

First, the result section is presented. Then, the discussion section is presented. In the result section, first, we derive the condition for cell quiescence. This condition brings up a non-dimensional number that we define as the inverse of potential-to-proliferation-increase (IPPI). When ${\rm{IPPI}}\ne 1$, cells are not quiescent. Next, we present the effect of decreasing NADH/NAD+ ratio on cell proliferation when ${\rm{IPPI}} > 1$. Here, we also present evidence from other studies to show that the reduction of NADH/NAD+ ratio is a general mechanism found in both cancer and normal cells, through which cells increase their proliferation. In IPPI, glycolysis, oxidative phosphorylation, and pyruvate-to-lactate conversion reactions (i.e., aerobic glycolysis) are involved. We present evidence that oxidative phosphorylation and pyruvate-to-lactate conversion reaction play a synergistic role in increasing cell proliferation when NADH/NAD+ ratio decreases. Then, we return to cell quiescence and show that both oxidative phosphorylation and pyruvate-to-lactate conversion reaction are essential for attaining cell quiescence, and aerobic glycolysis works as an amplifier of oxidative phosphorylation. In the discussion section, we discuss how metabolism, cell cycle, and apoptosis are linked. Further, we discuss the implication of our study for solid tumors in hypoxic conditions and cancer cell stemness and how lactate production profoundly affects different aspects of solid tumors, causing cancer progression. Finally, the method section is presented.

## Results

### The condition of cell quiescence

Cancer cells have poor DNA damage response and are sensitive to drugs that cause DNA damage in these cells during cell cycle progression^10^. On the other hand, cancer stem cells exit the cell cycle and are quiescent, causing drug resistance^11–13^. Further, normal cells, during differentiation, exit the cell cycle and become quiescent^14^. Furthermore, quiescence is a property of stem cells, preventing their functional exhaustion^15^. Below, we derive the condition for cell quiescence:

From Eq. 15 (Methods section), at the baseline and steady state,

1 $$\frac{{{\rm{k}}}_{3}}{{{\rm{k}}}_{4}+{{\rm{C}}}_{{\rm{P}}0}}-{{\rm{k}}}_{{\rm{d}}2}{{\rm{N}}}_{0}=0$$

where, ${{\rm{k}}}_{3}$ is NADH flux from glycolysis and ${{\rm{k}}}_{4}$ is the fold decrease in this flux due to the pyruvate-to-lactate conversion reaction. ${{\rm{N}}}_{0}$ is the baseline NADH concentration in the cytoplasm and ${{\rm{C}}}_{{\rm{P}}0}$ is cell proliferation, which is defined as the fold increase in the number of cells from a single cell, at the baseline.

Thus, from Eq. 1,

2 $$\frac{{{\rm{k}}}_{3}}{{{\rm{k}}}_{4}{{\rm{k}}}_{{\rm{d}}2}{{\rm{N}}}_{0}}=\frac{{{\rm{k}}}_{4}+{{\rm{C}}}_{{\rm{P}}0}}{{{\rm{k}}}_{4}}\ge 1$$

The equality in $\frac{{{\rm{k}}}_{4}+{{\rm{C}}}_{{\rm{P}}0}}{{{\rm{k}}}_{4}}\ge 1$ in Eq. 2 is when ${{\rm{C}}}_{{\rm{P}}0}=0$, and cells are quiescent at the baseline. Thus, for the quiescent cells at the baseline, the ratio ${\rm{IPPI}}=\frac{{{\rm{k}}}_{3}}{{{\rm{k}}}_{4}{{\rm{k}}}_{{\rm{d}}2}{{\rm{N}}}_{0}}=1$.

### A decrease in NADH/NAD+ ratio increases the cell proliferation

**Fig. 2:**
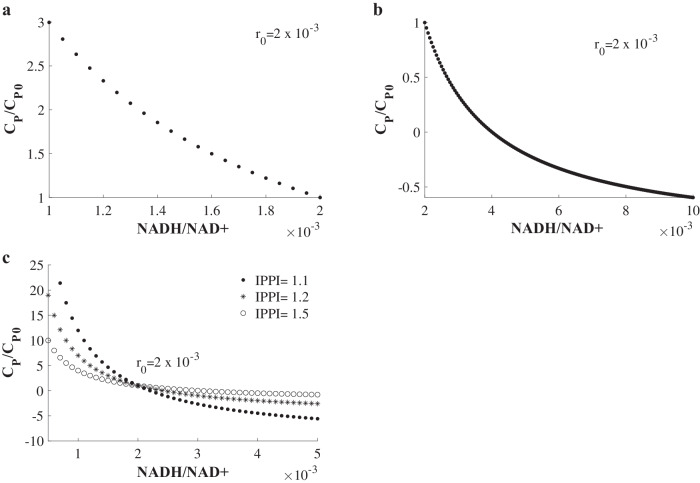
Effect of variation in NADH/NAD+ ratio on the relative cell proliferation. **a** The variation in the relative cell proliferation (C_P_/C_P0_) as a function of NADH/NAD+ ratio for IPPI = 2.0, when NADH/NAD+ ratio, r, is less than r_0_, which is the baseline NADH/NAD+ ratio. **b** The variation in the relative cell proliferation (C_P_/C_P0_) as a function of NADH/NAD+ ratio for IPPI = 2.0, when NADH/NAD+ ratio, r, is more than r_0_, which is the baseline NADH/NAD+ ratio. **c** The variation in the relative cell proliferation (C_P_/C_P0_) when NADH/NAD+ ratio, r, is varied for different values of the ratio ${\rm{IPPI}}=\frac{{{\rm{k}}}_{3}}{{{\rm{k}}}_{4}{{\rm{k}}}_{{\rm{d}}2}{{\rm{N}}}_{0}}$.

Our model shows that a decrease in NADH/NAD+ ratio, which may happen due to changes in the kinetic parameters of the cell cycle and apoptosis according to Eq. 16 (Methods section), increases cell proliferation (Fig. 2a) according to Eq. 18 (Methods section). Thus, the cancer cells may reset their NADH/NAD+ ratio to attain a higher proliferation rate.

The above conclusion is in agreement with experimental findings in both cancer and normal cells. For example, in cancer cells, which are usually in a high glycolytic state, the NADH/NAD+ ratio is low^4^. In this context, SRC activity reduces the NADH/NAD+ ratio by activating NADH ubiquinone-oxidoreductase^16^. Notably, SRC is well known to cause cell proliferation^17,18^. Further, SRC activity has been implicated in many types of cancers^19,20^, implicating the reduction of the NADH/NAD+ ratio in cancers.

On the other hand, in the normal fetal liver hematopoietic stem cells (FL-HSCs), the MDH1-mediated malate-aspartate NADH shuttle reduces the cytoplasmic NADH/NAD+ ratio^21^ and causes rapid proliferation of the cells^21^.

Further, memory characteristics of innate immunity protect against secondary infection after primary exposure to infections or vaccinations^22^. Monocytes trained with the β-glucan component of *Candida albicans* upregulated glycolysis and lactate production^22^ and decreased the NADH/NAD+ ratio. Interestingly, β-glucan from *Grifola frondosa* caused the proliferation of macrophages^23^, and cell cycle activation of β–glucan–trained cells has been observed^24^. Further, β-glucan induces the proliferation and activation of monocytes^25^. Thus, in general, a decrease in NADH/NAD+ ratio has been implicated in increasing the proliferation rate of cells. Therefore, the reduction in NADH/NAD+ ratio is a general mechanism to attain a higher cell proliferation through aerobic glycolysis (Fig. 2a).

In contrast, if the NADH/NAD+ ratio increases, cell proliferation decreases (Fig. 2b). In this context, SRC kinase phosphorylates the mitochondrial NADH ubiquinone-oxidoreductase, preserving its activity^16^. On the other hand, the inhibition of SRC blocks this activity^16^. Further, blocking the activity of NADH ubiquinone-oxidoreductase increases the cytoplasmic NADH/NAD+ ratio by reducing the NADH shuttles, MAS and G3PS, to mitochondria. Thus, the inhibition of SRC reduces the proliferation of cells by increasing the NADH/NAD+ ratio in cells.

Similarly, Optic Atrophy 1 (OPA1), which regulates cristae shape in mitochondria, controls the TCA cycle in T helper 17 (T_H_17) cells^26^. In agreement with the antagonistic effect of the increase in NADH/NAD+ ratio on cell proliferation, OPA1 deletion increased NADH/NAD+ ratio in T_H_17 cells, impaired IL-17 production, and decreased their proliferation^26^.

Moreover, increasing the NADH/NAD+ ratio further can cause cell proliferation to turn negative (Fig. 2b). Therefore, increasing the NADH/NAD+ ratio may be useful in anti-cancer therapy. Interestingly, the growth of the cells, whose proliferation increases more when the NADH/NAD+ ratio is decreased relative to the baseline NADH/NAD+ ratio r_0_, reduces faster and turns more negative when the NADH/NAD+ ratio is increased relative to the baseline NADH/NAD+ ratio r_0_ (Fig. 2c). Thus, the cells with a lower value of the ratio IPPI, $\frac{{{\rm{k}}}_{3}}{{{\rm{k}}}_{4}{{\rm{k}}}_{{\rm{d}}2}{{\rm{N}}}_{0}}$, in Eq. 18 (Methods section), are more amenable to anti-cancer therapy that increases their NADH/NAD+ ratio (Fig. 2c).

### The effect of ${\rm{IPPI}}=\frac{{{\rm{k}}}_{3}}{{{\rm{k}}}_{4}{{\rm{k}}}_{{\rm{d}}2}{{\rm{N}}}_{0}}$ ratio when IPPI ≠ 1

**Fig. 3:**
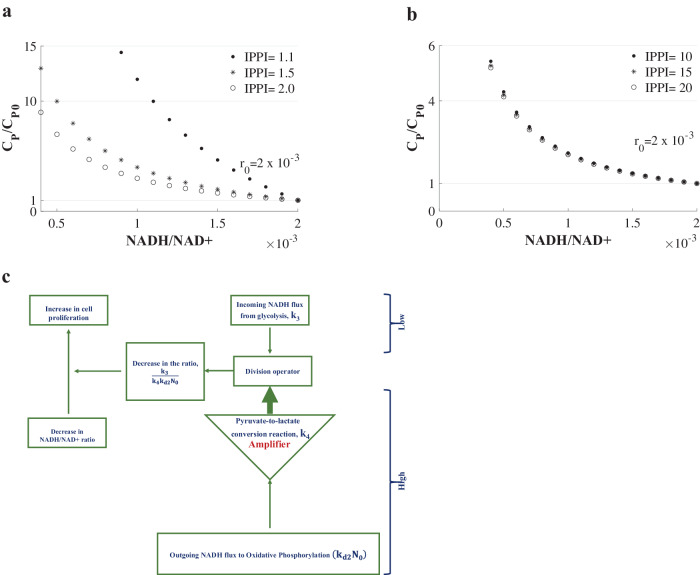
A decrease in NADH/NAD+ ratio increases the relative cell proliferation more when the ratio IPPI is lower and comparable to unity, for IPPI > 1. **a** The vari**a**tion in the relative cell proliferation (C_P_/C_P0_) when NADH/NAD+ ratio, r, is varied for different values of the ratio ${\rm{IPPI}}=\frac{{{\rm{k}}}_{3}}{{{\rm{k}}}_{4}{{\rm{k}}}_{{\rm{d}}2}{{\rm{N}}}_{0}}$ for r < r_0_ i.e. for a decrease in the NADH/NAD+ ratio. The ratio, IPPI, is comparable to unity. **b** The variation in the relative cell proliferation (C_P_/C_P0_) when NADH/NAD+ ratio, r, is varied for different values of the ratio ${\rm{IPPI}}=\frac{{{\rm{k}}}_{3}}{{{\rm{k}}}_{4}{{\rm{k}}}_{{\rm{d}}2}{{\rm{N}}}_{0}}$ for r < r_0_ i.e. for a decrease in the NADH/NAD+ ratio. The ratio, IPPI, is much higher than unity. **c** Pyruvate-to-lactate conversion reaction works as an amplifier of oxidative phosphorylation in increasing the relative proliferation rate when the NADH/NAD+ ratio decreases.

When the ratio IPPI, $\frac{{{\rm{k}}}_{3}}{{{\rm{k}}}_{4}{{\rm{k}}}_{{\rm{d}}2}{{\rm{N}}}_{0}}$, which is higher than unity according to inequality 2, is comparable to unity, the increase in cell proliferation, $\frac{{{\rm{C}}}_{{\rm{P}}}}{{{\rm{C}}}_{{\rm{P}}0}}$, due to a reduction in NADH/NAD+ ratio, depends on the $\frac{{{\rm{k}}}_{3}}{{{\rm{k}}}_{4}{{\rm{k}}}_{{\rm{d}}2}{{\rm{N}}}_{0}}$ (Fig. 3a). In contrast, if ${\rm{IPPI}}=\frac{{{\rm{k}}}_{3}}{{{\rm{k}}}_{4}{{\rm{k}}}_{{\rm{d}}2}{{\rm{N}}}_{0}}\gg 1$, the $\frac{{{\rm{C}}}_{{\rm{P}}}}{{{\rm{C}}}_{{\rm{P}}0}}$ is independent of the ratio $\frac{{{\rm{k}}}_{3}}{{{\rm{k}}}_{4}{{\rm{k}}}_{{\rm{d}}2}{{\rm{N}}}_{0}}$ (Fig. 3b). In the subsections below, we analyze the effect of different variables in the ratio $\frac{{{\rm{k}}}_{3}}{{{\rm{k}}}_{4}{{\rm{k}}}_{{\rm{d}}2}{{\rm{N}}}_{0}}$ on cell proliferation when the ratio, $\frac{{{\rm{k}}}_{3}}{{{\rm{k}}}_{4}{{\rm{k}}}_{{\rm{d}}2}{{\rm{N}}}_{0}}$, is comparable to unity.

#### A lower effectiveness in increasing the NADH concentration by glycolysis increases cell proliferation more when the NADH/NAD+ ratio decreases

The variable k_3_, from Eq. 15 (Methods section), is the incoming NADH flux from the upstream steps of glycolysis. From Fig. 3a, the increase in cell proliferation, when NADH/NAD+ ratio decreases, is higher for the lower values of IPPI, $\frac{{{\rm{k}}}_{3}}{{{\rm{k}}}_{4}{{\rm{k}}}_{{\rm{d}}2}{{\rm{N}}}_{0}}$. Thus, a lower value of k_3_ increases the proliferation more when the NADH/NAD+ ratio decreases. This is consistent with the increase in the activity of GAPDH by NAD+ when the effectiveness of increasing the cytoplasmic NADH concentration decreases due to a lower value of k_3_.

#### Pyruvate-to-lactate conversion reaction helps achieve a higher cell proliferation when the NADH/NAD+ ratio decreases

Since a lower magnitude of the non-dimensional number IPPI, $\frac{{{\rm{k}}}_{3}}{{{\rm{k}}}_{4}{{\rm{k}}}_{{\rm{d}}2}{{\rm{N}}}_{0}}$, increases the proliferation more (Fig. 3a), a higher magnitude of k_4_, which is the fold decrease in NADH flux due to pyruvate-to-lactate conversion reaction, increases the proliferation more when NADH/NAD+ decreases (Fig. 3a). Thus, the pyruvate-to-lactate conversion reaction or the Warburg effect helps cells attain a higher cell proliferation by decreasing their NADH/NAD+ ratio.

#### The flow of the reducing power toward oxidative phosphorylation increases the proliferation more when the NADH/NAD+ ratio decreases

Similarly, in the ratio $\frac{{{\rm{k}}}_{3}}{{{\rm{k}}}_{4}{{\rm{k}}}_{{\rm{d}}2}{{\rm{N}}}_{0}}$, a higher value of the variable k_d2_, which, according to Eq. 15 (Methods section), is related to the flux of NADH and NADH-equivalent toward oxidative phosphorylation and TCA cycle, also increases cell proliferation more when NADH/NAD+ decreases according to Fig. 3a.

Thus, the upstream steps in glycolysis, which increase NADH, are unhelpful in promoting relative cell proliferation, while the downstream steps of pyruvate-to-lactate conversion and oxidative phosphorylation, which consume NADH, promote the relative increase in cell proliferation. Further, the cytoplasmic NADH is detrimental to cell proliferation even though it causes cell cycle progression through CtBP.

In the ratio IPPI, $\frac{{{\rm{k}}}_{3}}{{{\rm{k}}}_{4}{{\rm{k}}}_{{\rm{d}}2}{{\rm{N}}}_{0}}$, the term ${{\rm{k}}}_{{\rm{d}}2}{{\rm{N}}}_{0}$ is the flux of NADH and NADH-equivalent to mitochondria (Eq. 15, Methods section). This flux is amplified by k_4_, the fold decrease in the incoming NADH flux from glycolysis due to the pyruvate-to-lactate conversion reaction, and the higher value of ${{\rm{k}}}_{4}{{\rm{k}}}_{{\rm{d}}2}{{\rm{N}}}_{0}$ increases the proliferation rate more when NADH/NAD+ ratio decreases (Fig. 3a and c). Thus, the pyruvate-to-lactate conversion reaction serves as an amplifier of oxidative phosphorylation in promoting relative cell proliferation (Fig. 3c).

### Cell quiescence requires a higher level of oxidative phosphorylation and/or aerobic glycolysis

From Eq. 17 (Methods section), for the cell quiescence at the baseline (i.e. C_P0_ = 0), the NADH concentration is:

3 $${{\rm{N}}}_{0}=\frac{{{\rm{k}}}_{3}}{{{\rm{k}}}_{{\rm{d}}2}{{\rm{k}}}_{4}}$$

Further, from Eq. 3, for the quiescence at the baseline, either the oxidative phosphorylation (k_d2_) or the lactate production (k_4_) should be high so that quiescence can be attained at a moderate level of NADH and not at an exorbitant level of NADH, which can cause apoptosis in the cells.

If k_d2_ = 0 or k_4_ = 0, i.e., if the cells hadn’t had the oxidative phosphorylation or the lactate production, from Eq. 3, to achieve quiescence, the cell would need an infinite concentration of NADH. Thus, in the absence of oxygen or pyruvate-to-lactate conversion reaction, the cell will never achieve quiescence. In agreement, it has been found that respiration is essential for maintaining the quiescence of adult hematopoietic stem cells^27^. Further, in agreement with the above analysis, the overexpression of LDH in muscle stem cells causes their quiescence^28^. In contrast, isocitrate dehydrogenase (IDH) mutation in glioma causes lactate dehydrogenase LDH-A silencing^29^ and the glioma cells do not attain quiescence^30^. Interestingly, IDH mutant gliomas have a better prognosis^29^ since they are not quiescent, and quiescence is related to the stemness of cancer cells. In contrast, gliomas that do not have IDH mutation express higher levels of PROM1 and SOX2, the stem cell markers^31^. PROM1 expression causes quiescence in glioma cells^32^. Thus, LDH-A activity in IDH-wild-type gliomas is involved in conferring stemness and quiescence by increasing lactate production (k_4_). Therefore, aerobic glycolysis is a general mechanism that also incorporates the attainment of quiescence.

The above analysis is for the quiescence of the cells at the baseline when Eq. 18 (Methods section) is not applicable because its denominator becomes zero, and instead, we used Eq. 17 (Methods section). On the other hand, if the baseline cells are proliferating, Eq. 18 is applicable. For the quiescence of these cells (i.e. C_P_ = 0) at the NADH/NAD+ ratio of r, from Eq. 18 (Methods section),

4 $$\left(\frac{{{\rm{k}}}_{3}}{{{\rm{k}}}_{4}{{\rm{k}}}_{{\rm{d}}2}{{\rm{N}}}_{0}}\right)\left(\frac{{\rm{r}}+1}{{{\rm{r}}}_{0}+1}\right)\frac{{{\rm{r}}}_{0}}{{\rm{r}}}-1=0$$

where, ${{\rm{r}}}_{0}$ is the baseline NADH/NAD+ ratio.

Since in eukaryotic cells ${\rm{r}}\,\ll\, 1$ and ${{\rm{r}}}_{0}\,\ll\, 1$^33^,

$$\frac{{\rm{r}}+1}{{{\rm{r}}}_{0}+1}\approx 1$$

Then, from Eq. 4, to attain quiescence at an NADH/NAD+ ratio of r

5 $$\frac{{\rm{r}}}{{{\rm{r}}}_{0}}\approx \frac{{{\rm{k}}}_{3}}{{{\rm{k}}}_{4}{{\rm{k}}}_{{\rm{d}}2}{{\rm{N}}}_{0}}$$

From Eq. 5 and inequality 2, for attaining quiescence at r by the cells whose baseline is proliferating

6 $$\frac{{\rm{r}}}{{{\rm{r}}}_{0}}=\frac{{{\rm{k}}}_{3}}{{{\rm{k}}}_{4}{{\rm{k}}}_{{\rm{d}}2}{{\rm{N}}}_{0}} > 1$$

Thus, from inequality 6, for the quiescence of the proliferating cells, since the ratio, $\frac{{\rm{r}}}{{{\rm{r}}}_{0}}$, should be higher than unity, the proliferating cells have to increase their NADH/NAD+ ratio to achieve quiescence. In contrast, cancer cells decrease their NADH/NAD+ ratio, although the hypoxic environment of solid tumors brings near quiescence in cancer cells by increasing the NADH/NAD+ ratio^34^.

### Aerobic glycolysis works as an amplifier of oxidative phosphorylation to maintain a high level of NAD+ in cells

From Eq. 6, for quiescence (Supplementary Information 1 and Supplementary Movie 1)

7 $${{\rm{k}}}_{3}=\left({{\rm{k}}}_{4}\right).\left({{\rm{k}}}_{{\rm{d}}2}{\rm{N}}\right)$$

or

8 $$\begin{array}{ll}{\rm{Incoming}}\,{\rm{NADH}}\,{\rm{flux}}\,{\rm{from}}\,{\rm{glycolysis}} = {\rm{product}}\, {\rm{of}}\, {\rm{the}}\, {\rm{fold}}\, {\rm{reduction}}\, {\rm{in}}\, {\rm{the}}\, {\rm{incoming}}\, {\rm{NADH}}\, {\rm{flux}}\, {\rm{from}}\, {\rm{glycolysis}}\, {\rm{due}}\, {\rm{to}}\, {\rm{the}}\,{\rm{pyruvate}}{\hbox{-}}{\rm{to}}{\hbox{-}}{\rm{lactate}}\,{\rm{conversion}}\, {\rm{reaction}}\,\left({\rm{k}}_{4}\right)\,{\rm{and}}\, {\rm{the}}\, {\rm{outgoing}}\, {\rm{flux}}\, {\rm{of}}\, {\rm{NADH}}\, {\rm{to}}\, {\rm{oxidative}}\, {\rm{phosphorylation}}\,\left({\rm{k}}_{{\rm{d}}2}{\rm{N}}\right)\end{array}$$

**Fig. 4:**
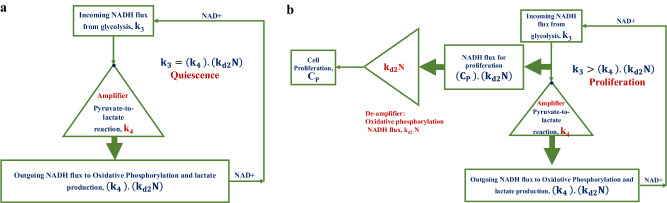
The conditions for cell quiescence and cell proliferation. **a** The condition, ${{\rm{k}}}_{3}={{\rm{k}}}_{4}{{\rm{k}}}_{{\rm{d}}2}{{\rm{N}}}_{0}$, is satisfied for the cell quiescence. Further, the pyruvate-to-lactate conversion reaction works as an amplifier in maintaining the high concentration of NAD+ in cells. **b** The condition, ${{\rm{k}}}_{3} >\, {{\rm{k}}}_{4}{{\rm{k}}}_{{\rm{d}}2}{{\rm{N}}}_{0}$, is satisfied for cell proliferation. The difference, ${{\rm{k}}}_{3}-{{\rm{k}}}_{4}{{\rm{k}}}_{{\rm{d}}2}{{\rm{N}}}_{0}$, is the NADH flux for cell proliferation through CtBP. This flux is de-amplified by the NADH flux for the oxidative phosphorylation to give the cell proliferation C_P_.

In Eq. 7 or 8, the outgoing NADH flux to oxidative phosphorylation (k_d2_ N) is amplified by the lactate production reaction (k_4_) (Fig. 4a). Thus, the lactate production reaction works as an amplifier to maintain a high concentration of NAD+ in cells (Fig. 4a).

On the other hand, for the proliferative cells, from Eq. 18 (Methods section),

9 $$\left(\frac{{{\rm{k}}}_{3}}{{{\rm{k}}}_{4}{{\rm{k}}}_{{\rm{d}}2}{{\rm{N}}}_{0}}\right)\left(\frac{{\rm{r}}+1}{{{\rm{r}}}_{0}+1}\right)\frac{{{\rm{r}}}_{0}}{{\rm{r}}}-1\, >\, 0$$

Since, $$\frac{{\rm{r}}\,+\,1}{{{\rm{r}}}_{0}\,+\,1}\approx 1$$

From Eq. 9,

10 $$\frac{{\rm{r}}}{{{\rm{r}}}_{0}} < \frac{{{\rm{k}}}_{3}}{{{\rm{k}}}_{4}{{\rm{k}}}_{{\rm{d}}2}{{\rm{N}}}_{0}}$$

From Eq. 10 (Supplementary Information 2), for proliferating cells

11 $${{\rm{k}}}_{3} > \left({{\rm{k}}}_{4}\right).\left({{\rm{k}}}_{{\rm{d}}2}{\rm{N}}\right)$$

or

12 $$\begin{array}{ll}{\rm{Incoming}}\, {\rm{NADH}}\, {\rm{flux}}\, {\rm{from}}\, {\rm{glycolysis}}\, >\, {\rm{Product}}\, {\rm{of}}\, {\rm{the}}\, {\rm{fold}}\, {\rm{reduction}}\, {\rm{in}}\, {\rm{the}}\, {\rm{incoming}}\, {\rm{NADH}}\, {\rm{flux}}\, {\rm{from}}\, {\rm{glycolysis}}\, {\rm{due}}\, {\rm{to}}\, {\rm{the}}\,{\rm{pyruvate}}{\hbox{-}}{\rm{to}}{\hbox{-}}{\rm{lactate}}\, {\rm{conversion}}\, {\rm{reaction}}\,\left({\rm{k}}_{4}\right)\, {\rm{and}}\, {\rm{the}}\, {\rm{outgoing}}\, {\rm{flux}}\, {\rm{of}}\, {\rm{NADH}}\, {\rm{to}}\, {\rm{oxidative}}\,{\rm{phosphorylation}}\,\left({\rm{k}}_{{\rm{d}}2}{\rm{N}}\right)\end{array}$$

From inequalities 11 or 12, we infer that the extra incoming NADH flux from glycolysis is used for proliferation (Fig. 4b) through CtBP (Supplementary Movie 2).

From Eq. 15 (Methods section), at the steady state,

13 $$\left({{\rm{C}}}_{{\rm{P}}}\right).\left({{\rm{k}}}_{{\rm{d}}2}{\rm{N}}\right)={{\rm{k}}}_{3}-\left({{\rm{k}}}_{4}\right).\left({{\rm{k}}}_{{\rm{d}}2}{\rm{N}}\right)$$

Thus, the outgoing NADH flux for proliferation through CtBP is ${{\rm{C}}}_{{\rm{P}}}.\left({{\rm{k}}}_{{\rm{d}}2}{\rm{N}}\right)$ (Eq. 13 and Fig. 4b). Further, the cell proliferation C_P_ is obtained after the conversion of this flux into cell proliferation with the help of a de-amplifier, which is the NADH flux (k_d2_ N) to oxidative phosphorylation (Left-hand side of Eq. 13 and Fig. 4b).

Furthermore, since the effect of a decrease in NADH concentration is multiplied by k_d2_ and k_4_ (the term $\left({{\rm{k}}}_{4}\right).\left({{\rm{k}}}_{{\rm{d}}2}{\rm{N}}\right)$ in the right-hand side of Eq. 13), the effect of the decrease in NADH/NAD+ ratio in increasing the relative cell proliferation is promoted by both the oxidative phosphorylation and the pyruvate-to-lactate conversion reaction (Supplementary Movie 2). Moreover, the pyruvate-to-lactate conversion reaction amplifies the effect of oxidative phosphorylation in increasing the relative cell proliferation (Fig. 4b) when the NADH/NAD+ ratio decreases through the term, $\left({{\rm{k}}}_{4}\right).\left({{\rm{k}}}_{{\rm{d}}2}{\rm{N}}\right)$, in Eq. 13 (Supplementary Movie 2).

Further, although the outgoing NADH flux, $\left({{\rm{C}}}_{{\rm{P}}}\right).\left({{\rm{k}}}_{{\rm{d}}2}{\rm{N}}\right)$, for proliferation causes proliferation through CtBP, it is de-amplified by oxidative phosphorylation (Left-hand side of Eq. 13 and Fig. 4b). Thus, oxidative phosphorylation couples with the cell cycle progression caused by NADH-CtBP.

## Discussion

We found that higher rates of oxidative phosphorylation and aerobic glycolysis are required for attaining both a higher cell proliferation, when the NADH/NAD+ ratio decreases, and quiescence. Thus, the same cellular processes are required for both the progression of the cell cycle and the exit from it. Further, we found a non-dimensional number, which we define as the inverse of potential-to-proliferation-increase (IPPI), that regulates cell proliferation. If this number is unity, cells exit from the cell cycle and become quiescent. In contrast, if this number is higher than unity, cells proliferate. The lower magnitude of this number implies a higher proliferation rate. Our model and analysis elucidate the link between metabolism and cell cycle and may be important in understanding how cancer cells regulate their proliferation and devising strategies to increase the NADH/NAD+ ratio in these cells to reduce their proliferation and cause apoptosis in these cells.

NADH concentration is not an independent variable but is controlled by cell proliferation through the cell cycle and apoptosis (Eq. 16, Methods section) involving NADH-CtBP. Indeed, a decrease in the cytosolic NADH/NAD+ ratio decreases the activation of MAPK8 and BAX, inhibiting the apoptotic pathways^35^. In contrast, an increase in the NADH/NAD+ ratio has been shown to cause TP53 accumulation^4^ and a decrease in CDKN1A level^7^, causing apoptosis^9,36^. Thus, varying the NADH/NAD+ ratio in the cell affects the cell cycle and apoptosis and vice versa.

The environment within a solid tumor is hypoxic, which reduces oxidative phosphorylation or, in our analysis, reduces k_d2_ in Eq. 15 (Methods section), raising the term $\frac{{{\rm{k}}}_{3}}{{{\rm{k}}}_{4}{{\rm{k}}}_{{\rm{d}}2}{{\rm{N}}}_{0}}$ or the right-hand side of Eq. 6, $\frac{{\rm{r}}}{{{\rm{r}}}_{0}}=\frac{{{\rm{k}}}_{3}}{{{\rm{k}}}_{4}{{\rm{k}}}_{{\rm{d}}2}{{\rm{N}}}_{0}}$, required to be satisfied by the cells to attain quiescence, thus, attaining some degree of proliferation. Further, hypoxia has been shown to increase NADH/NAD+ ratio^34^, satisfying the condition $\frac{{\rm{r}}}{{{\rm{r}}}_{0}} > 1$ in inequality 6, thus, attaining a relative quiescence. Therefore, hypoxia creates a competition between proliferation and quiescence by increasing both sides of Eq. 6, $\frac{{\rm{r}}}{{{\rm{r}}}_{0}}=\frac{{{\rm{k}}}_{3}}{{{\rm{k}}}_{4}{{\rm{k}}}_{{\rm{d}}2}{{\rm{N}}}_{0}}$. Consequently, in the hypoxic zone, cells are proliferative but close to attaining quiescence. Further, the more severe the hypoxia, i.e., the less the oxidative phosphorylation (i.e., the lower the value of k_d2_), the more the value of k_4_ in Eq. 6, i.e., the higher may be the lactate production, to keep Eq. 6, $\frac{{\rm{r}}}{{{\rm{r}}}_{0}}=\frac{{{\rm{k}}}_{3}}{{{\rm{k}}}_{4}{{\rm{k}}}_{{\rm{d}}2}{{\rm{N}}}_{0}}$, nearly satisfied, and thus, the competition between proliferation and quiescence continues through an increase in both hypoxia and lactate production controlling the ratio $\frac{{{\rm{k}}}_{3}}{{{\rm{k}}}_{4}{{\rm{k}}}_{{\rm{d}}2}{{\rm{N}}}_{0}}$ and satisfying the condition $\frac{{\rm{r}}}{{{\rm{r}}}_{0}}=\frac{{{\rm{k}}}_{3}}{{{\rm{k}}}_{4}{{\rm{k}}}_{{\rm{d}}2}{{\rm{N}}}_{0}}$. Therefore, at the center, assumed at a point of highest hypoxia in the solid tumor, the lactate production may be the highest, and there may be a gradient of lactate from the presumed center to the surface.

Interestingly, since from Eq. 6 for the quiescence of proliferating cells the condition $\frac{{\rm{r}}}{{{\rm{r}}}_{0}}={\rm{IPPI}} > 1$ needs to be satisfied and since cells have a higher proliferation rate when IPPI is lower and close to unity, when IPPI is near 1, the cells under hypoxia are both highly proliferative (Fig. 3a) and closer to attaining quiescence because they have to minimally increase their NADH/NAD+ ratio to meet the condition $\frac{{\rm{r}}}{{{\rm{r}}}_{0}} > 1$ and satisfy the condition $\frac{{\rm{r}}}{{{\rm{r}}}_{0}}={\rm{IPPI}} > 1$ for attaining quiescence. Further, since hypoxia increases the NADH/NAD+ ratio^34^, the proliferative cells need to increase the level of hypoxia to attain quiescence. Thus, the cells at the presumed center of solid tumors, where hypoxia is the maximum, may be quiescent.

Further, the quiescence caused by hypoxic conditions may increase the stability of both cancer cells and hematopoietic stem cells residing in their respective hypoxic niche. Interestingly, the quiescence in hematopoietic stem cells is caused by TP53^37^, which accumulates due to increased NADH-CtBP^4^ under hypoxia as hypoxia increases the NADH/NAD+ ratio in cells^34^. Similarly, the quiescence in cancer cells is caused by TP53-responsive miRNAs, miRNA-27b-3p and miRNA-455-3p, which increase Cdk inhibitor CDKN1B levels^38^. On the other hand, CDKN1A, another Cdk inhibitor downstream of TP53, is redundant in causing cell quiescence^39^. Notably, we found that IPPI = 1 is a general condition for cell quiescence. Thus, regardless of how it is implemented by the cell cycle, cell quiescence is caused by metabolism, which creates the overriding condition for it.

Further, the lactate at the surface of the solid tumor causes angiogenesis by the lactate-mediated activation of the RAF-ERK pathway through an oxygen-regulated protein NDRG3^40^. Angiogenesis brings glucose and oxygen to the surface and inside of a solid tumor, and these two diffuse from outside to inside of the tumor. Further, the lactate at the surface creates an immunosuppressive environment surrounding the tumor^41^. Furthermore, the cells at the presumed center of the solid tumors have autophagy due to severe hypoxia^42,43^. Since quiescence and autophagy^44–46^ are properties of stem cells^47^, and hypoxia stimulates stemness and epithelial-to-mesenchymal transition^48–50^ along with quiescence and autophagy, these cells in a severe hypoxic zone around the presumed center may have a higher degree of stemness. Thus, the location of cells near the presumed center of a solid tumor may be partly responsible for their stemness.

Our study suggests that the pyruvate-to-lactate conversion reaction is an amplifier of oxidative phosphorylation (OXPHOS) and the Warburg effect may not be a specific phenomenon of cancer cells. The cells have two degrees of freedom through tuning between aerobic glycolysis and OXPHOS, lowering the NADH/NAD+ ratio, and achieving a higher proliferation rate. This suggestion is supported by observation in cell lines. For example, some cell lines exhibit both high oxygen-consuming rate (OCR) and extracellular acidification rate (ECAR), and display fast proliferation, such as the MCF7 cell line, while very few cell lines may display a similar fast cell proliferation but with relatively lower OCR and higher ECAR values such as PC3. In fast proliferative cells, the high glycolytic rate is coupled with high OXPHOS activity. In a simple model, the high glycolytic rate feeds abundant substrate to de-novo synthesis of nuclear acids and lipids, essential for producing new cells. The abundant NADH production from the TCA cycle powers the electron transport chain, pentose phosphate pathway, and lipid synthesis. On the other hand, under unfavorable conditions, such as mitochondrial dysfunction, even though some cancer cells can survive with limited mitochondrial functions, their proliferation speed can be significantly impaired.

In summary, the paper showed that a negative feedback loop (Methods section) is at the core of the Warburg effect and provided an in-depth insight into this effect. Further, our results suggest that cells in a hypoxic environment are highly proliferative yet close to attaining quiescence by increasing their NADH/NAD+ ratio through the increase in the intensity of hypoxia. Our model and analysis help us understand the role of aerobic glycolysis in cell proliferation, quiescence, and other important aspects of solid tumors.

## Methods

### The basis for a negative feedback loop between the rate of NADH production and the rate of increase in cell proliferation

NADH, through CtBP, represses CDKN1A and causes cell cycle progression. Thus, the rate of NADH-increase increases the cell proliferation rate through the cell cycle, working as the positive arm of the feedback loop (Fig. 1b). On the other hand, NAD+ is required for glycolysis, which is required for the continued increase in cell proliferation rate. Thus, a continued increase in cell proliferation rate requires higher and higher conversion of NADH to NAD + , reducing the rate of NADH increase and working as the negative arm of the loop (Fig. 1b).

### Model

The cytosolic/nuclear NADH regulates the cellular processes through the C-terminal binding protein (CtBP). CtBP is a global corepressor that forms repressor complexes with other corepressors in the presence of NADH^8,51,52^. CtBP represses CDKN1A^7^, a universal inhibitor of cyclin kinases^53^, causing cell cycle progression (Fig. 1a). Thus, cell proliferation increases with the increase in NADH concentration. The dependence of the rate of increase of cell proliferation, ${{\rm{C}}}_{{\rm{P}}}$, on NADH concentration, N, can be given by Hill’s function for a positive regulation. The positive regulation of the cell proliferation rate can also be given by the Michaelis-Menten kinetics in which the cell proliferation C_p_ works as an enzyme and NADH concentration, N, takes the place of a substrate. The growth rate also decreases due to apoptosis. Thus, the rate of change in cell proliferation, ${{\rm{C}}}_{{\rm{P}}}$, is given as:

14 $$\frac{{\rm{d}}{{\rm{C}}}_{{\rm{P}}}}{{\rm{dt}}}=\frac{{{\rm{k}}}_{1}{{\rm{C}}}_{{\rm{P}}}{\rm{N}}}{{{\rm{k}}}_{2}+{\rm{N}}}-{{\rm{k}}}_{{\rm{d}}1}{{\rm{C}}}_{{\rm{P}}}$$

where, k_1_, k_2_, and k_d1_ are parameters, C_P_ is the cell proliferation, and N is the NADH concentration. Cell proliferation C_P_ is defined as the fold increase in the number of cells from a single cell. The parameter k_1_ represents the NADH-dependent cell division caused by the repression of CDKN1A by CtBP-NADH. The parameter k_2_ represents NADH-independent apoptosis, while the addition of N in the denominator ${{\rm{k}}}_{2}+{\rm{N}}$ is due to the NADH-dependent apoptosis caused by the accumulation of TP53 and the decrease in CDKN1A by NADH. Further, as a normal regulatory mechanism in cells, an increase in the proliferation rate is countered by the cell cycle-dependent apoptosis caused by the tumor-suppressor genes such as TP53 and RB1^54^. The parameter k_d1_ represents the cell cycle-dependent apoptosis. The parameter k_2_ represents the basal level of apoptosis. It can be measured when NADH concentration in the cell is not externally altered. On the other hand, NADH concentration in the cell can be increased by inhibiting NADH oxidases and apoptosis can be measured. Thus, the proportionality constant between an increase in NADH concentration and the rate of apoptosis can be measured for calculating the value of ${{\rm{k}}}_{2}+{\rm{N}}$ in the denominator of Eq. 14. Similarly, the rate of cell cycle progression can be increased and the rate of apoptosis caused by the increase in cell cycle can be calculated to find the value of the constant k_d1_.

Equation 14 is based on the precondition that all cells will conduct mitotic division, generating identical progeny cells after each mitotic cycle, which is true among normal stem cells. However, malignant cells exhibit disordered mitotic cycles, i.e., non-canonical mitosis, such as entering endocycles, yielding polyploid cells containing multiple copies of the genome with a simultaneously enlarged cell body. These cells may retain a similar NADH concentration in Eq. 14. However, Cp is the fold increase in the cell number from a single cell. In the case of endocycles, we need to define cell mass Cm, which is the fold increase in the cell mass from a single cell mass. Cm is proportional to Cp. We will have to determine the proportionality constant to convert Cp into Cm and vice versa.

Glycolysis produces NADH, increasing cell proliferation through CtBP and CDKN1A (Fig. 1a). On the other hand, NADH inhibits continued glycolysis by inhibiting GAPDH. Further, the onward oxidation of NADH to generate lactate helps unblock glycolysis through the pyruvate-to-lactate conversion reaction, which decreases NADH. This decrease lowers the overall rate of increase of NADH with aerobic glycolysis. Thus, a higher and higher cell proliferation causes a lower and lower increase in NADH, i.e., a continued increase in cell proliferation gives a diminishing return of the rate of increase of NADH in the cytoplasm. Therefore, there is a negative regulation of the rate of increase of NADH in the cells by the cell proliferation, C_p_.

We denote the NADH flux from glycolysis as ${{\rm{k}}}_{3}$ and fold decrease in this flux due to pyruvate-to-lactate conversion reaction as ${{\rm{k}}}_{4}$. This fold decrease is augmented by dilution in the NADH concentration due to cell proliferation ${{\rm{C}}}_{{\rm{P}}}$, which is defined as the fold increase in the number of cells from a single cell. Thus, the rate of increase in NADH concentration, by taking into account the glycolysis, the pyruvate-to-lactate conversion reaction, and the dilution due to the cell proliferation, is given as $\frac{{{\rm{k}}}_{3}}{{{\rm{k}}}_{4}+{{\rm{C}}}_{{\rm{P}}}}$, which is also the Hill’s function for the negative regulation of the NADH rate by cell proliferation (Fig. 1b). Further, the flux of NADH and NADH-equivalent is also diverted to mitochondria for oxidative phosphorylation and TCA cycle, respectively. We assume that the flux of NADH and NADH-equivalent to mitochondria follows a first-order kinetics and is, thus, given as ${{\rm{k}}}_{{\rm{d}}2}{\rm{N}}$, where $\frac{1}{{{\rm{k}}}_{{\rm{d}}2}}$ is the resistance of the NADH and NADH-equivalent flux to mitochondria. Thus, the rate of change of NADH concentration in the cytoplasm by taking into account the generation of NADH in the cytoplasm and removal from the cytoplasm is given as:

15 $$\frac{{\rm{dN}}}{{\rm{dt}}}=\frac{{{\rm{k}}}_{3}}{{{\rm{k}}}_{4}+{{\rm{C}}}_{{\rm{P}}}}-{{\rm{k}}}_{{\rm{d}}2}{\rm{N}}$$

where, k_d2_ is a parameter.

The positive regulation of the proliferation rate by NADH-CtBP and negative regulation of NADH by the cell proliferation makes a negative feedback loop between the cell proliferation rate and the rate of NADH increase (Fig. 1b). This negative feedback loop is the key to resolving the Warburg effect.

From Eq. 14, at the steady state,

16 $${\rm{N}}=\frac{{{\rm{k}}}_{{\rm{d}}1}{{\rm{k}}}_{2}}{{{\rm{k}}}_{1}-{{\rm{k}}}_{{\rm{d}}1}}$$

Thus, NADH concentration in the cytoplasm is controlled by the parameters of the cell cycle and apoptosis.

Further, from Eq. 15, at the steady state,

17 $${{\rm{C}}}_{{\rm{P}}}=\frac{{{\rm{k}}}_{3}}{{{\rm{k}}}_{{\rm{d}}2}{\rm{N}}}-{{\rm{k}}}_{4}$$

Thus, an increase in the NADH concentration decreases cell proliferation, a consequence of the negative feedback loop between cell proliferation and NADH concentration in the cytoplasm (Fig. 1b).

Let ${\rm{r}}=\frac{{\rm{NADH}}}{{{\rm{NAD}}}^{+}}$, then from Eq. 17 at the steady state (Supplementary Information 3),

18 $$\frac{{{\rm{C}}}_{{\rm{P}}}}{{{\rm{C}}}_{{\rm{P}}0}}=\frac{\left(\frac{{{\rm{k}}}_{3}}{{{\rm{k}}}_{4}{{\rm{k}}}_{{\rm{d}}2}{{\rm{N}}}_{0}}\right)\left(\frac{{\rm{r}}\,+\,1}{{{\rm{r}}}_{0}\,+\,1}\right)\frac{{{\rm{r}}}_{0}}{{\rm{r}}}-1}{\left(\frac{{{\rm{k}}}_{3}}{{{\rm{k}}}_{4}{{\rm{k}}}_{{\rm{d}}2}{{\rm{N}}}_{0}}\right)-1}$$

where ${{\rm{C}}}_{{\rm{P}}0}$ is the cell proliferation at the NADH/NAD+ ratio of ${{\rm{r}}}_{0}$ when NADH concentration is ${{\rm{N}}}_{0}$ (the baseline) and ${{\rm{C}}}_{{\rm{P}}}$ is the cell proliferation when the NADH/NAD+ ratio is ${\rm{r}}$.

We denote the ratio $\frac{{{\rm{k}}}_{3}}{{{\rm{k}}}_{4}{{\rm{k}}}_{{\rm{d}}2}{{\rm{N}}}_{0}}$ as the inverse-of-the potential-to-proliferation-increase (IPPI). Thus,

19 $${\rm{IPPI}}=\frac{{{\rm{k}}}_{3}}{{{\rm{k}}}_{4}{{\rm{k}}}_{{\rm{d}}2}{{\rm{N}}}_{0}}$$

From Eq. 17, an increase in k_4_, the fold decrease in the incoming NADH flux from glycolysis due to the pyruvate-to-lactate conversion reaction, decreases cell proliferation. Similarly, an increase in k_d2_, representing oxidative phosphorylation, decreases the proliferation, while an increase in k_3_, representing the increase in NADH flux due to the upstream steps in glycolysis, increases cell proliferation. Thus, based on this basic underlying motif (Fig. 1b), on an absolute basis, the pyruvate-to-lactate conversion reaction and oxidative phosphorylation do not help promote cell proliferation if they were not required to provide metabolites for the anabolic reactions required for cell proliferation because the cytoplasmic NADH through CtBP can cause the cell cycle progression, suggesting that the presence of mitochondria is not helpful for an organism’s proliferation.

However, Eq. 17 also shows that a decrease in the NADH concentration, N increases the cell proliferation. Thus, on a relative basis, the processes that help reduce the NADH concentration in the cytoplasm can help increase cells’ proliferation. In agreement, from Eq. 18, on a relative basis, the pyruvate-to-lactate conversion reaction and oxidative phosphorylation help cells attain a higher cell proliferation (the results section) when the NADH/NAD+ ratio decreases. Thus, carcinogenesis may use aerobic glycolysis to attain a higher, relative cell proliferation through a decrease in NADH/NAD+ ratio while oxidative phosphorylation plays a synergistic role.

### The parameters of the model

The only important parameter in this model is in the form of a non-dimensional number, $\frac{{{\rm{k}}}_{3}}{{{\rm{k}}}_{4}{{\rm{k}}}_{{\rm{d}}2}{{\rm{N}}}_{0}}$, which is denoted as the inverse-of-the potential-to-proliferation-increase (IPPI) (Figs. 2c and 3a, b).

From Eq. 17, the cells are quiescent (C_P0_ = 0) for IPPI = 1. Further, when IPPI > 1, cells have a positive proliferation ${{\rm{C}}}_{{\rm{P}}0}\, >\, 0$ from Eq. 17. In contrast, when IPPI < 1, the cells have a negative proliferation ${{\rm{C}}}_{{\rm{P}}0}\, <\, 0$ from Eq. 17.

When IPPI > 1, i.e., when the baseline proliferation is positive, and when ${\rm{r}}\,\ll\, 1$ and ${{\rm{r}}}_{0}\,\ll\, 1$ (NADH/NAD+ ratio varies from 1/700 to 1/60^33^ in eukaryotic cells),

20 $$\left(\frac{{\rm{r}}+1}{{{\rm{r}}}_{0}+1}\right)\approx 1$$

then, as ${\rm{r}}$ decreases, i.e., ${\rm{r}}\, <\, {{\rm{r}}}_{0}$, ${{\rm{C}}}_{{\rm{P}}}\, >\, {{\rm{C}}}_{{\rm{P}}0}$ from Eq. 18. Thus, from Eq. 18, for IPPI > 1 and ${{\rm{r}}}_{0}\,\ll\, 1$, as NADH/NAD+ ratio decreases, the cell proliferation increases above the baseline value.

In contrast, when ${\rm{r}}\,\gg\, 1$ and ${{\rm{r}}}_{0}\,\gg\, 1$, $\left(\frac{{\rm{r}}\,+\,1}{{{\rm{r}}}_{0}\,+\,1}\right)\frac{{{\rm{r}}}_{0}}{{\rm{r}}}=1$ in Eq. 18. Thus, $\frac{{C}_{{\rm{P}}}}{{{\rm{C}}}_{{\rm{P}}0}}=1$, from Eq. 18, when ${\rm{r}}\gg 1$ and ${{\rm{r}}}_{0}\,\gg\, 1$. Thus, when NADH is much higher than NAD+ in the cells, the cells’ proliferation is invariant. Therefore, in the result section, we only considered the physically realistic case of ${\rm{r}}\,\ll\, 1$ and ${{\rm{r}}}_{0}\,\ll\, 1$. Further, we only considered the case when ${\rm{IPPI}}\ge 1$ because the baseline cell proliferation is non-negative under this condition. Thus, our results above are generally valid for ${\rm{r}}\,\ll\, 1$ and ${{\rm{r}}}_{0}\,\ll\, 1$, and ${\rm{IPPI}}\ge 1$.

Under the above conditions, as shown in the result section, a lower value of IPPI is more potent in increasing the relative cell proliferation, i.e., in increasing the ratio, $\frac{{{\rm{C}}}_{{\rm{P}}}}{{{\rm{C}}}_{{\rm{P}}0}}$, when the NADH/NAD+ ratio decreases. Further, since IPPI is a ratio of the process that increases NADH (i.e., k_3_ or the incoming NADH flux from glycolysis) to the processes that decrease NADH (i.e., the product of k_4_ or the fold decrease in the incoming NADH flux from glycolysis due to the pyruvate-to-lactate conversion reaction and k_d2_. N or the outgoing NADH flux to oxidative phosphorylation) and since a lower value of IPPI is more potent in increasing the cell proliferation (the results section), the retention of the reducing power of NADH in the cytoplasm is detrimental to the increase in the relative cell proliferation. Below, we define the dimensionless parameter, IPPI, as:

21 $${\rm{IPPI}}=\frac{{\rm{Incoming\; NADH\; flux\; from\; glycolysis}}\left({k}_{3}\right)}{\begin{array}{c}{\rm{Product\; of\; the\; fold\; reduction\; in\; the\; incoming\; NADH\; flux\; from\; glycolysis\; due\; to}}\,\\ {\rm{pyruvate}}-{\rm{to}}-{\rm{lactate\; conversion\; reaction}}\left({{\rm{k}}}_{4}\right){\rm{and\; the\; outgoing\; NADH\; flux\; to}}\\ {\rm{oxidative\; phosphorylation}}\left({{\rm{k}}}_{{\rm{d}}2}{{\rm{N}}}_{0}\right)\end{array}}$$

### Reporting summary

Further information on research design is available in the Nature Research Reporting Summary linked to this article.

### Supplementary information

Supplementary Information

Reporting summary

Supplementary Movie 1. The condition for cell quiescence.

Supplementary Movie 2: The role of glycolysis, pyruvate-to-lactate conversion reaction, and oxidative phosphorylation in decreasing the NADH/NAD+ ratio and causing cell proliferation.

## Data Availability

All data supporting the findings of this study are available within the paper and its Supplementary Information.

## Code availability

Computer code will be made available upon request.
