# Supplementary material for: A negative feedback loop underlies the Warburg effect
Source: NPJ Syst Biol Appl. 2024 May 24;10:55. doi: 10.1038/s41540-024-00377-x (PMC11126737; doi:10.1038/s41540-024-00377-x)
Supplement: Supplementary file 1 — Supplementary Information [file 41540_2024_377_MOESM1_ESM.pdf]

Supplementary Information 1

At the baseline, the NADH and NAD+ concentrations are N_0 and A_0, respectively, and the NADH/NAD+ ratio is r_0. Then, X moles of NADH convert to NAD+, changing the NADH/NAD+ ratio to r and NADH and NAD+ concentrations to N and A, respectively.

Thus, from equation 6 in the main manuscript

$$\frac{\mathrm{N}/A}{N_0/A_0} = \frac{\mathrm{k_3}}{\mathrm{k_4 k_{d2} N_0}}\,, \tag{1}$$

or,

$$\frac{N}{A/A_0} = \frac{\mathrm{k_3}}{\mathrm{k_4 k_{d2}}}\,, \tag{2}$$

Since,

$$A = A_0 + X, \tag{3}$$

or,

$$A/A_0 = 1 + \frac{X}{A_0}\,, \tag{4}$$

Since the concentration of NAD+ in the eukaryotic cells is large in comparison to the NADH concentration[1],

$$\frac{X}{A_0} \approx 0, \tag{5}$$

Thus,

$$A/A_0 \approx 1, \tag{6}$$

Therefore, from equation 2,

$$\mathrm{k_3} = \mathrm{k_4 k_{d2}} N$$

Supplementary Information 2

At the baseline, NADH and NAD+ concentrations are N_0 and A_0, respectively, and NADH/NAD+ ratio is r_0. Then, X moles of NADH convert to NAD+, changing the NADH/NAD+ ratio to r and NADH and NAD+ concentrations to N and A, respectively.

From equation 10 in the main manuscript

$$\frac{\mathrm{N}/A}{N_0/A_0} < \frac{\mathrm{k_3}}{\mathrm{k_4 k_{d2} N_0}}, \qquad (1)$$

or,

$$\frac{N}{A/A_0} < \frac{\mathrm{k_3}}{\mathrm{k_4 k_{d2}}}, \qquad (2)$$

Since,

$$A = A_0 + X, \qquad (3)$$

or,

$$A/A_0 = 1 + \frac{X}{A_0}, \qquad (4)$$

Since the concentration of NAD+ in the eukaryotic cells is large in comparison to the NADH concentration[1],

$$\frac{X}{A_0} \approx 0, \qquad (5)$$

Thus,

$$A/A_0 \approx 1, \qquad (6)$$

Therefore, from equation 2

$$\mathrm{k_3} > \mathrm{k_4 k_{d2}} N$$

Supplementary Information 3

At the baseline, the concentrations of NADH and NAD+ are N_0 and A_0, respectively. At the baseline, the cell proliferation is C_{P0}, and the NADH/NAD+ ratio is r_0. Then, X moles of NADH convert to NAD+, changing the NADH/NAD+ ratio to r and cell proliferation to C_P. Thus, the concentration of NADH becomes $N=N_0 - X$, and the concentration of NAD+ becomes $A=A_0 + X$.

$$N = N_0 - X, \quad (1)$$

$$A = A_0 + X, \quad (2)$$

$$r = \frac{N}{A_0+X}, \quad (3)$$

From equations 1 and 3,

$$r = \frac{N}{A_0+N_0-N}, \quad (4)$$

or,

$$N = \frac{r(A_0+N_0)}{(r+1)}, \quad (5)$$

From equation 17 in the main manuscript,

$$C_{P0} = \frac{k_3}{k_{d2}\, N_0} - k_4, \quad (6)$$

and

$$C_P = \frac{k_3}{k_{d2}\, N} - k_4, \quad (7)$$

Thus, from equations 6 and 7

$$\frac{C_P}{C_{P0}} = \left(\frac{k_3 - k_4\, k_{d2}\, N}{k_{d2}\, N}\right)\left(\frac{k_{d2}\, N_0}{k_3 - k_4\, k_{d2}\, N_0}\right), \quad (8)$$

or,

$$\frac{C_P}{C_{P0}} = \left(\frac{N_0}{N}\right)\left(\frac{k_3 - k_4\, k_{d2}\, N}{k_3 - k_4\, k_{d2}\, N_0}\right), \quad (9)$$

From equations 5 and 9,

$$\frac{C_P}{C_{P0}} = \frac{N_0(r+1)}{r(N_0 + A_0)} \left(\frac{k_3 - \frac{k_4\, k_{d2}\, r(N_0 + A_0)}{r+1}}{k_3 - k_4\, k_{d2}\, N_0}\right), \quad (10)$$

or,

$$\frac{C_P}{C_{P0}} = \frac{N_0}{r(N_0 + A_0)} \left(\frac{k_3(r+1) - k_4\, k_{d2}\, r(N_0 + A_0)}{k_3 - k_4\, k_{d2}\, N_0}\right), \tag{11}$$

or,

$$\frac{C_P}{C_{P0}} = \left(\frac{\frac{k_3\ (r+1)}{r\,(N_0 + A_0)\, k_4\, k_{d2}} - 1}{\frac{k_3}{k_4\, k_{d2}\ N_0} - 1}\right), \tag{12}$$

or,

$$\frac{C_P}{C_{P0}} = \left(\frac{\frac{k_3\left(1 + \frac{1}{r}\right)}{\left(1 + \frac{1}{r_0}\right) k_4\, k_{d2}\, N_0} - 1}{\frac{k_3}{k_4\, k_{d2}\ N_0} - 1}\right), \tag{13}$$

or,

$$\frac{C_P}{C_{P0}} = \left(\frac{\frac{k_3}{k_4\, k_{d2}\, N_0}\ \frac{(r+1)}{(r_0+1)}\ \frac{r_0}{r} - 1}{\frac{k_3}{k_4\, k_{d2}\ N_0} - 1}\right)$$

References

1. Yang, Y. & Sauve, A. A. NAD+ metabolism: Bioenergetics, signaling and manipulation for therapy. *Biochim Biophys Acta* **1864**, 1787 (2016).
